# Effects of exercise interventions on executive function in autism spectrum disorder: a three-level meta-analytic review

**DOI:** 10.3389/fpsyt.2026.1780503

**Published:** 2026-07-20

**Authors:** Jiangdi Su, Liang Li, Yuchen Wang, Tonggang Fan

**Affiliations:** College of Wushu, Shanghai University of Sport, Shanghai, China

**Keywords:** autism spectrum disorder, children and adolescents, executive function, exercise intervention, three-level meta-analysis

## Abstract

**Objective:**

This study aimed to use a three-level meta-analysis to examine the effects of exercise interventions on executive function (EF) in children and adolescents with autism spectrum disorder (ASD), and to explore the relationship between exercise dose and intervention effects through subgroup analyses.

**Methods:**

A systematic search was conducted in Web of Science, PubMed, Embase, and the Cochrane Library from database inception to November 17, 2025. All included studies were randomized controlled trials. A three-level random-effects model was applied to integrate multiple dependent effect sizes. Hedges’ g was used to calculate effect sizes, and negative values indicated improvements in EF following exercise interventions. Study quality was assessed using the PEDro scale, and publication bias was evaluated using Egger’s regression and the trim-and-fill method.

**Results:**

Seventeen studies involving 626 participants were included. Exercise interventions produced a moderate and significant improvement in EF (*g* = –0.34, *p* < 0.0001) with low heterogeneity (*Q = 50.42, p =0.268*). Subgroup analyses showed that only intervention duration significantly moderated the effects, with programs lasting ≥10 weeks yielding the greatest benefits.

**Conclusion:**

Exercise interventions significantly improve EF in children and adolescents with ASD, with the largest effect sizes observed in interventions lasting ≥10 weeks. Further large-scale studies are needed to clarify dose–response relationships and inform optimal exercise prescriptions. This meta-analysis was designed and reported in accordance with the Preferred Reporting Items for Systematic Reviews and Meta-Analyses (PRISMA 2020) guidelines.

**Systematic review registration:**

https://www.crd.york.ac.uk/PROSPERO/, identifier CRD420251236112

## 1 Introduction

ASD is a neurodevelopmental disorder characterized by impaired social interaction, communication difficulties, and repetitive or stereotyped behaviors (1). Its prevalence has increased steadily over the past two decades. Long-term monitoring data from the US Autism and Developmental Disabilities Monitoring program indicate that the prevalence of ASD among eight-year-olds increased from 0.67% in 2000 to 2.78% in 2020 (2), representing more than a threefold increase. This upward trend imposes substantial burdens on educational systems, families, and public health services (3).

EF is a higher-order cognitive process that involves the purposeful regulation of thoughts and behaviors to achieve specific goals and encompasses inhibitory control, working memory, and cognitive flexibility (4). Among the various functional impairments associated with ASD, deficits in EF are considered a core cognitive difficulty, particularly in these domains (5–8). Impaired EF not only exacerbates difficulties in social communication and repetitive behaviors (9, 10), but also restricts participation in physical activities, emotional regulation, and daily adaptation (11, 12). These deficits collectively undermine the overall development and quality of life of children and adolescents with ASD (13).

Currently, common non-pharmacological interventions for executive dysfunction in children with ASD primarily include EF task training (14, 15), dietary modifications (16), and exercise interventions (17, 18). Among these, exercise interventions have increasingly become a primary approach for enhancing EF in children with ASD because of their diverse formats, low implementation costs, and favorable safety profile.

Although existing meta-analyses have generally concluded that exercise interventions exert positive effects on overall EF, findings across specific EF subdomains remain inconsistent. For instance, significant intervention effects were observed only for inhibitory control and cognitive flexibility, with no improvement in working memory (19, 20). Conversely, several previous meta-analyses reported improvement trends across multiple EF subdomains; however, effect sizes varied considerably between subdomains (21, 22). Notably, several previous studies conducted subgroup analyses based on intervention dosage (20, 23, 24); however, no study has performed a meta-regression analysis to examine the dose–response relationship. Furthermore, conventional two-level meta-analytic methods were predominantly employed in prior research, which inadequately accounted for statistical dependencies among multiple effect sizes within the same study. This limitation resulted in statistical bias and consequently compromised the accuracy of effect size estimates.

In this context, three-level meta-analysis provides an appropriate statistical framework for addressing common challenges related to multiple outcomes, shared samples, and correlated effect sizes in ASD exercise intervention studies. By simultaneously estimating variance at the study level, the within-study outcome level, and the sampling level, this approach prevents information loss that may occur when effect sizes are merged or selectively discarded and yields more accurate and robust pooled estimates (25). Accordingly, the present study aims to systematically evaluate the overall improvement in EF among children and adolescents with ASD following exercise interventions using a three-level meta-analytic framework. The specific objectives are to calculate the combined effect size of exercise interventions relative to control conditions in enhancing overall EF and to examine the moderating effects of key exercise prescription characteristics, including intervention duration, frequency, exercise session time, and total intervention time, through subgroup and meta-regression analyses, thereby providing evidence-based guidance for optimizing exercise interventions for ASD.

## 2 Methods

### 2.1 Protocol and registration

This meta-analysis was designed and reported in accordance with the Preferred Reporting Items for Systematic Reviews and Meta-Analyses (PRISMA 2020) guidelines. The review protocol was prospectively registered in the International Prospective Register of Systematic Reviews (registration ID: CRD420251236112).

### 2.2 Search strategy

This study followed established systematic review procedures. Two reviewers independently conducted comprehensive searches of PubMed, the Cochrane Library, Embase, and Web of Science from database inception to November 17, 2025. The search strategy integrated Medical Subject Headings (MeSH) and free-text terms, with core keywords including “exercise,” “autism spectrum disorder”, “Executive Function”, “Child”, and “Randomized Controlled Trial”, combined using Boolean operators (AND, OR). Search terms were refined and adapted to the indexing characteristics of each database. In addition, the reference lists of included studies and relevant reviews were screened using a snowballing approach to identify potentially eligible studies not captured in the initial search. The full electronic search strategy for each database is provided in the Appendix.

### 2.3 Selection criteria

This study applied inclusion criteria grounded in the PICOS framework (26): (1) Population—children and adolescents (<18 years) formally diagnosed with ASD according to the DSM, ICD, or other internationally recognized criteria, without restrictions on sex or geographic region; (2) Intervention—structured exercise or exercise-based programs in which physical activity constituted the primary therapeutic component, regardless of type, intensity, frequency, or duration. Programs incorporating auxiliary elements (e.g., music, games, or virtual/interactive contexts) were eligible only when these served as supportive delivery formats rather than independent therapeutic components; (3) Comparator—no-intervention controls, routine activities, or other non-exercise conditions, with multi-arm trials included if at least one comparison involved exercise versus an eligible control; (4) Outcomes—at least one objective, standardized measure of EF, including inhibitory control (e.g., Stroop, Go/No-Go, Flanker), working memory (e.g., N-back, digit span), or cognitive flexibility (e.g., WCST, TMT); and (5) Study design—randomized controlled trials reporting sufficient statistical data (e.g., means, standard deviations, and sample sizes) to calculate effect sizes.

The exclusion criteria were as follows: (1) studies involving mixed populations without separately reported ASD subgroup data or participants outside the child and adolescent age range without stratified results; (2) studies in which the exercise component could not be clearly identified or sufficiently described, as well as multicomponent interventions in which exercise was not the primary active ingredient (e.g., psychotherapy- or music therapy-led programs involving only incidental physical activity), or in which the exercise condition did not substantially differ from the control condition; (3) studies that did not report objective, standardized, and quantifiable EF measures or lacked essential statistical data for effect size calculation that could not be obtained from the authors; and (4) non-randomized studies, animal experiments, conference abstracts without full text, duplicate publications, studies with missing key results, or those without accessible full texts.

### 2.4 Data extraction

This study was conducted by two independent reviewers (LL and WY), who screened the literature, extracted data, and cross-checked all information according to the predefined inclusion and exclusion criteria. Any discrepancies were resolved through discussion or, when necessary, by consultation with a third reviewer (SJ). The extracted data included basic study information (title, first author, publication year, and country), participant characteristics (age and sample size), details of the intervention and control conditions (intervention duration, frequency, exercise session time, and total intervention time), and outcome measures with their corresponding results.

In addition, based on authoritative guidelines such as the Physical Activity Guidelines for Americans and the American College of Sports Medicine (27, 28), as well as previous systematic reviews (29, 30) and the intervention descriptions reported in the included studies, this study classified physical activity (PA) interventions into four categories. The definitions of each PA intervention category are presented in **Table 1**.

**Table 1:** Treatment nodes included in the meta-analysis.

Node	Definition
Exergaming	Exergaming refers to interactive video game based interventions that promote physical activity by requiring body movements during gameplay, combining exercise with entertainment, such as Nintendo Wii and Xbox Kinect (28).
Aerobic Exercise (AE)	Aerobic exercise refers to endurance based activities involving large muscle groups performed continuously or rhythmically to improve cardiorespiratory fitness, such as brisk walking, running, and cycling (26,27).
Mind-body Exercise (MBE)	Mind body exercise refers to activities that combine physical movement with mental focus, emphasizing breathing control, posture, and rhythm, to promote both physical and psychological regulation, such as yoga and tai chi (29).
Multicomponent Physical Activity (MPA)	Multicomponent physical activity refers to activities that combine different movement elements such as motor skills, coordination, and balance with continuous physical activity, while involving cognitive engagement, such as leaning cycling, and racket sports.

### 2.5 Outcome extraction and coding

The selection of EF outcomes was independently conducted by two reviewers (LL and WY). To ensure comparability across outcome measures and avoid disproportionate weighting of individual samples, a unified rule was applied for extracting EF indicators. When a study reported only an EF composite score, this composite score was extracted as the sole effect size and entered into the three-level meta-analysis. When both composite and subdomain outcomes (e.g., inhibitory control, cognitive flexibility, or working memory) were reported, only subdomain-specific effect sizes were retained, and the composite score was excluded to prevent information redundancy and artificial inflation of study weight. Furthermore, during subgroup analyses of EF subdomains, effect sizes reflecting only overall EF outcomes without specific subdomain classification were excluded to improve the interpretability and accuracy of the subdomain-level analyses. Any discrepancies between the two reviewers were resolved through discussion or, when necessary, through consultation with a third reviewer (JS).

### 2.6 Data analysis

Because the included studies frequently reported multiple EF outcomes, effect sizes derived from the same study were statistically dependent, and the use of a conventional two-level random-effects model could underestimate standard errors and inflate statistical significance. To appropriately address this dependency, a three-level random-effects meta-analytic model was adopted in accordance with the framework proposed by Assink and Wibbelink, which partitions variance into sampling error (level 1), within-study variation among multiple outcomes (level 2), and between-study heterogeneity (level 3) (31). The three-level model was implemented using established open-source R code (32, 33), and all analyses were conducted in R (version 4.4.2) using the metafor package with restricted maximum likelihood estimation (34).

Effect sizes and corresponding variances were computed in Microsoft Excel using pre- and post-test means, standard deviations, and sample sizes extracted from the included studies, in accordance with previously validated calculation methods (32, 33). Hedges’ g was used as the effect size metric, with directional standardization and small-sample correction applied such that negative values indicated greater EF improvement in the intervention group. Prior to estimating overall effects, influence analyses were conducted to identify potential outliers. Heterogeneity was assessed using multilevel Q statistics and stratified I^2^ indices, and likelihood ratio tests (LRTs) were used to examine the significance of variance components. Three-level mixed-effects moderator analyses were performed to examine intervention characteristics, and conventional two-level random-effects models that ignored effect size dependency were additionally estimated as robustness checks.

### 2.7 Publication bias and sensitivity analysis

To systematically evaluate the potential impact of small-sample effects and publication bias, this study adopted a multi-method assessment strategy. First, funnel plots were generated to visually inspect the symmetry of effect size distributions. Given that the included meta-analytic dataset contained multiple correlated effect sizes per study, Egger’s regression tests were performed under both two-level and three-level specifications to detect small-sample effects more rigorously. In parallel, the trim-and-fill procedure—adapted for multilevel meta-analytic structures—was applied to estimate potentially missing studies and to derive bias-adjusted pooled effect sizes. To avoid distortion of funnel plot patterns and ensure the validity of regression-based tests, all publication bias analyses were conducted after excluding anomalous effect sizes identified during influence diagnostics.

### 2.8 Risk of bias and quality of evidence

Risk of bias for the included RCTs was evaluated using the Physical Therapy Evidence Database (PEDro) scale. This tool comprises 10 methodological items, including protocol adherence, randomization, allocation concealment, baseline comparability, control of exercise load, blinding of outcome assessment, dropout rate ≤ 15%, intention-to-treat analysis, appropriate between-group statistical comparisons, and reporting of point estimates with measures of variability. Each item was scored as 1 (criterion met) or 0 (criterion not met), yielding a maximum possible score of 10. Studies scoring < 4 were categorized as low quality, scores of 4–5 as moderate quality, 6–8 as fair quality, and 9–10 as high quality. Two researchers (LL and WJ) independently assessed study quality, and any disagreements were resolved through consultation with a third researcher (SJ), who provided the final judgment.

## 3 Result

### 3.1 Literature search and screening process

**Figure 1:**
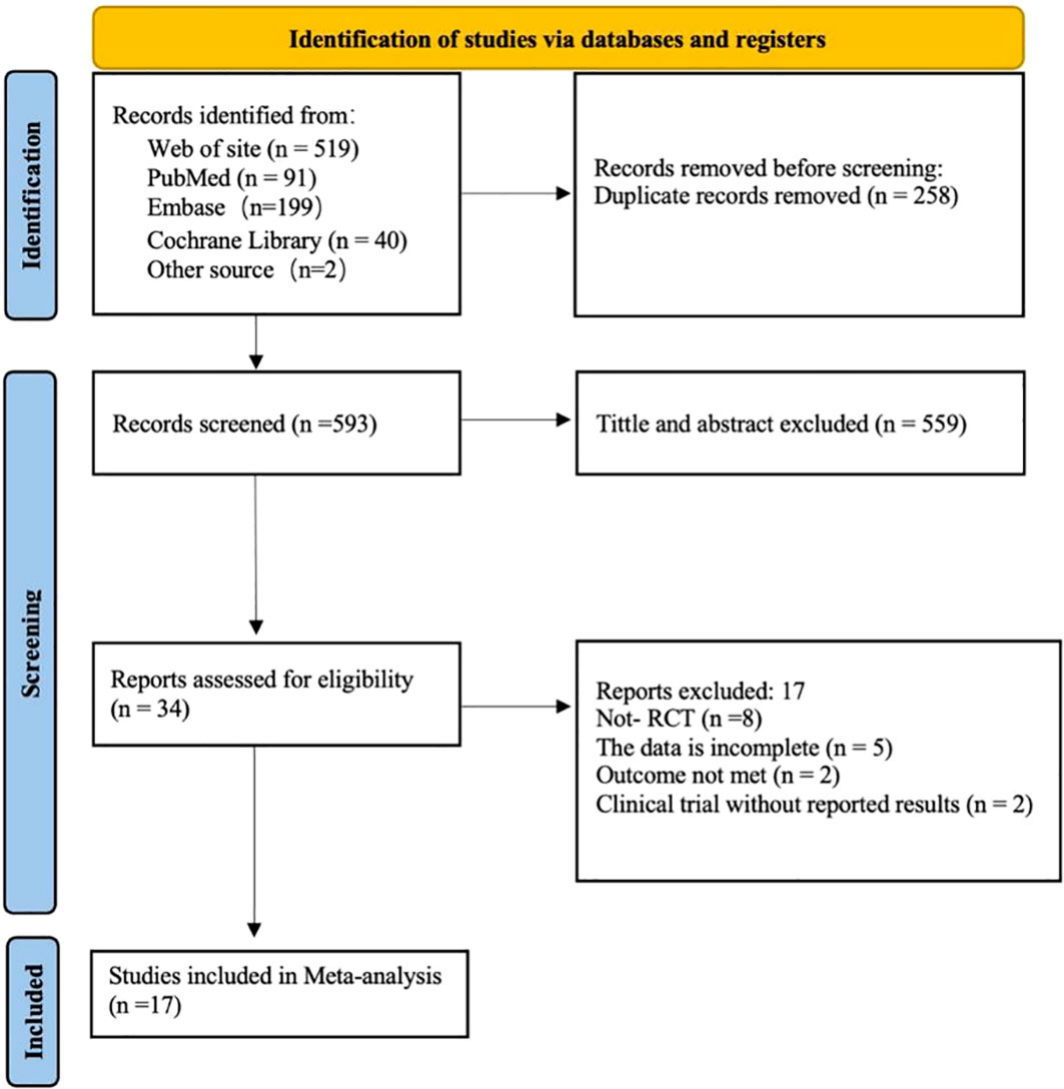
PRISMA flow diagram for studies retrieved through the electronic search and selection processes.

**Figure 1** presents the detailed process of literature identification and study selection.

A total of 851 records were initially retrieved from Web of Science (n = 519), PubMed (n = 91), Embase (n = 199), the Cochrane Library (n = 40), and additional sources (n = 2). After removing 258 duplicates, 593 records were screened by title and abstract, resulting in the exclusion of 559 articles that were unrelated to the research question. Full-text assessment was conducted for the remaining 34 articles, of which 17 were excluded for reasons including non-randomized study design (n = 8), non-eligible outcome measures (n = 2), insufficient data for effect size calculation (n = 5), and clinical trials without reported results (n = 2). Ultimately, 17 studies met all eligibility criteria and were included in the three-level meta-analysis.

### 3.2 Characteristics of included studies

This meta-analysis included 17 RCTs (35–51). The characteristics of the included studies are presented in **Table 2**. The study samples originated from China, Iran, Australia, Italy, South Korea, the United States, Egypt, and Taiwan, China, with a total sample size of 626 participants (342 in the experimental group and 284 in the control group). Participants were predominantly aged 3–12 years and were diagnosed with ASD using standardized tools, including DSM-5, DSM-IV-TR, ADOS-2, ADI-R, or GARS-3. Intervention types were diverse and encompassed traditional sports (table tennis, basketball, gymnastics, karate), mind–body exercises (yoga, Neiyang Gong), exergames, integrated physical activities, sensory integration training, mixed martial arts, and FMS training. Prescribed intervention sessions typically lasted 30–70 minutes and occurred 2–3 times per week over 4–12 weeks, with a few programs extending to 18 weeks. EF was assessed using standardized tools, including Go/No-Go, Stroop, WCST, Digit Span, and BRIEF/BRIEF-2. Most studies did not include follow-up assessments.

**Table 2:** Characteristics of the included studies.

Study,country	Age range	Diagnostic methods	Sample size (EG,CG)	Male (%)	Type	Nude	Time	Frequency	Duration	Intensity	Outcome	Follow-up time
Alooche,Iran	6–12	GARS-3	15/15	50%	Physical activity program	MPA	45	3	8	/	BRIEF, WCST, Digit span	/
Chan,China	6–17	DSM-IV-TR、ADI-R	20/20	90%	Nei Yang Gong	MBE	60	2	4	/	CCTT-2	/
Chenliang Deng,China	6–9	DSM-5	12/12	50%	Gymnastics	MPA	40	3	12	Moderate	Day-Night Stroop Task, Self-Ordered Pointing Task, Adapted WCST	/
Chien Yu Pan,Taiwan	6—12	DSM-IV-TR	11/11	100.00%	Table tennis	MPA	70	3	12	/	WCST	/
Gianpiero Greco,Italy	8–11	ADOS-2	14/14	85.7%	Karate	Exergame	45	2	12	/	BRIEF	/
Hao Chen,China	3–6	DSM-5 ADOS-2	15/15	83.3%	Sports game	MPA	30	6	8	Moderate	Digital Span Task, Day/Night Stroop Task, Dimensional Change Card Sorting	/
Junchen Deng,China	4–11	ADI-R	9/9	77.8%	Sensory Integration Training	MPA	60	3	8	50–69%	Go/No-Go Task	/
Lindor,Australia	7—12	DSM-5	14/13	48.15%	Dance	MBE	60	1	10	/	BRIEF-2	NO
Neka,Korean	6–18	Clinical ASD Diagnosis according to institutional evaluation	12/12	91.67%	Exergame	Exergame	30	2	4	/	Digit Span Forward, WCST , Stroop	/
Phung,USA	8–11	ADOS-2	14/20	82.35%	Mixed Martial Arts	MBE	45	2	13	/	Hearts & Flowers test, BRIEF-2	/
Qiang Wang,China	/	Clinical diagnosis	12/10	92%	Fundamental Movement Skill training	MPA	45	4	18	60–90%	BRIEF-2	/
Sepehri Bonab, Iran	7–10	GARS-3、DSM-5	20/20	100%	Exergame	Exergame	30	2	8	/	WCST, Flanker	/
Rafiei Milajerdi,Iran	6—10	ADOS-2	20/20/20	95.00%	Exergame/Spark	Exergame/MPA	35	3	8	/	WCST	/
Tanksale,Australia	8–12	/	31/30	63.93%	Yoga	MBE	60	1	6	/	BRIEF-2	6week
Tes,Egpt	8–12	DSM-5	19/21	80%	Basketball	MPA	45	2	12	/	GNG, BDS	/
Tes,Egpt	8–12	DSM-5 ADOS-2	22/20/20	80.65%	Learning bicycle、Stationary cycling	MPA/AE	60	5	2	/	SCWT, GNG, BDS	/
Tes,Egpt	8–12	DSM-5 ADOS-2	23/19/22	67.74%	Learning bicycle、Stationary cycling	MPA/AE	60	5	2	/	SCWT, GNG	/

BRIEF, Behavior Rating Inventory of Executive Function; BRIEF-2, Behavior Rating Inventory of Executive Function – Second Edition; WCST, Wisconsin Card Sorting Test; Adapted WCST, Adapted Wisconsin Card Sorting Test; CCTT-2, Children’s Color Trails Test – Second Edition; Stroop/SCWT, Stroop Color–Word Test; Self-Ordered Pointing Task, Self-Ordered Pointing Task; Dimensional Change Card Sorting, Dimensional Change Card Sort Task; Hearts & Flowers Test, Hearts and Flowers Task; Flanker, Eriksen Flanker Task; BDS, Backward Digit Span; AE, aerobic exercise; MBE, mind–body exercise; MPA, multicomponent physical activity.

### 3.3 Quality assessment

The 17 included RCTs scored between 5 and 8 on the PEDro scale, with most clustering around 6, indicating overall moderate-to-good methodological quality. Specifically, three studies were rated as moderate quality (4–5 points), 12 as good quality (6–7 points), and two as high quality (8–10 points) (**Table 3**). All studies implemented randomization, ensured baseline comparability, and reported between-group statistical comparisons. However, allocation concealment was achieved in only seven studies, and participant blinding was reported in just one study. No study employed therapist blinding, whereas assessor blinding was documented in six studies, suggesting a potential risk of performance and detection bias. Most studies adequately addressed attrition (16/17), and 10 applied intention-to-treat analysis, thereby strengthening the reliability of the findings.

**Table 3:** Methodological quality of included RCTs assessed by the PEDro scale.

Study	Randomization allocation	Allocation concealment	Similar at baseline	Subject blinded	Therapist blinded	Assessor blinded	Dropout rate	Intention-to-treat analysis	Between-group comparison	Point measures	Total score
Alooche 2025 Iran (40)	√	×	√	×	×	×	√	√	√	√	6
Chan 2013 China (35)	√	×	√	×	×	√	√	√	√	√	7
Chenliang Deng 2025 China (37)	√	√	√	×	×	√	√	√	√	√	8
Chien Yu Pan 2017 Taiwan (43)	√	×	√	×	×	×	√	√	√	√	6
Gianpiero Greco 2020 Italy (39)	√	√	√	×	×	×	√	√	√	√	7
Hao Chen 2024 China (36)	√	√	√	×	×	×	√	√	√	√	7
Junchen Deng 2023 China (38)	√	×	√	×	×	×	√	×	√	√	5
Lindor 2023 Australia (41)	√	√	√	×	×	√	×	×	√	√	6
Nekar 2022 Korean (42)	√	√	√	√	×	×	√	√	√	√	8
Phung 2023 USA (44)	√	×	√	×	×	×	√	√	√	√	6
Qiang Wang 2025 China (51)	√	×	√	×	×	×	√	×	√	√	5
Sepehri Bonab 2024 Iran (47)	√	×	√	×	×	×	√	√	√	√	6
Rafiei Milajerdi 2021 Iran (46)	√	×	√	×	×	×	√	√	√	√	6
Tanksale 2021 Australia (45)	√	×	√	×	×	×	√	×	√	√	5
Tes 2019 Egypt (50)	√	×	√	×	×	√	√	×	√	√	6
Tes 2021 Egypt (48)	√	√	√	×	×	√	√	×	√	√	7
Tes 2023 Egypt (49)	√	√	√	×	×	√	√	×	√	√	7

### 3.4 The effect of exercise interventions on EF

**Figure 2:**
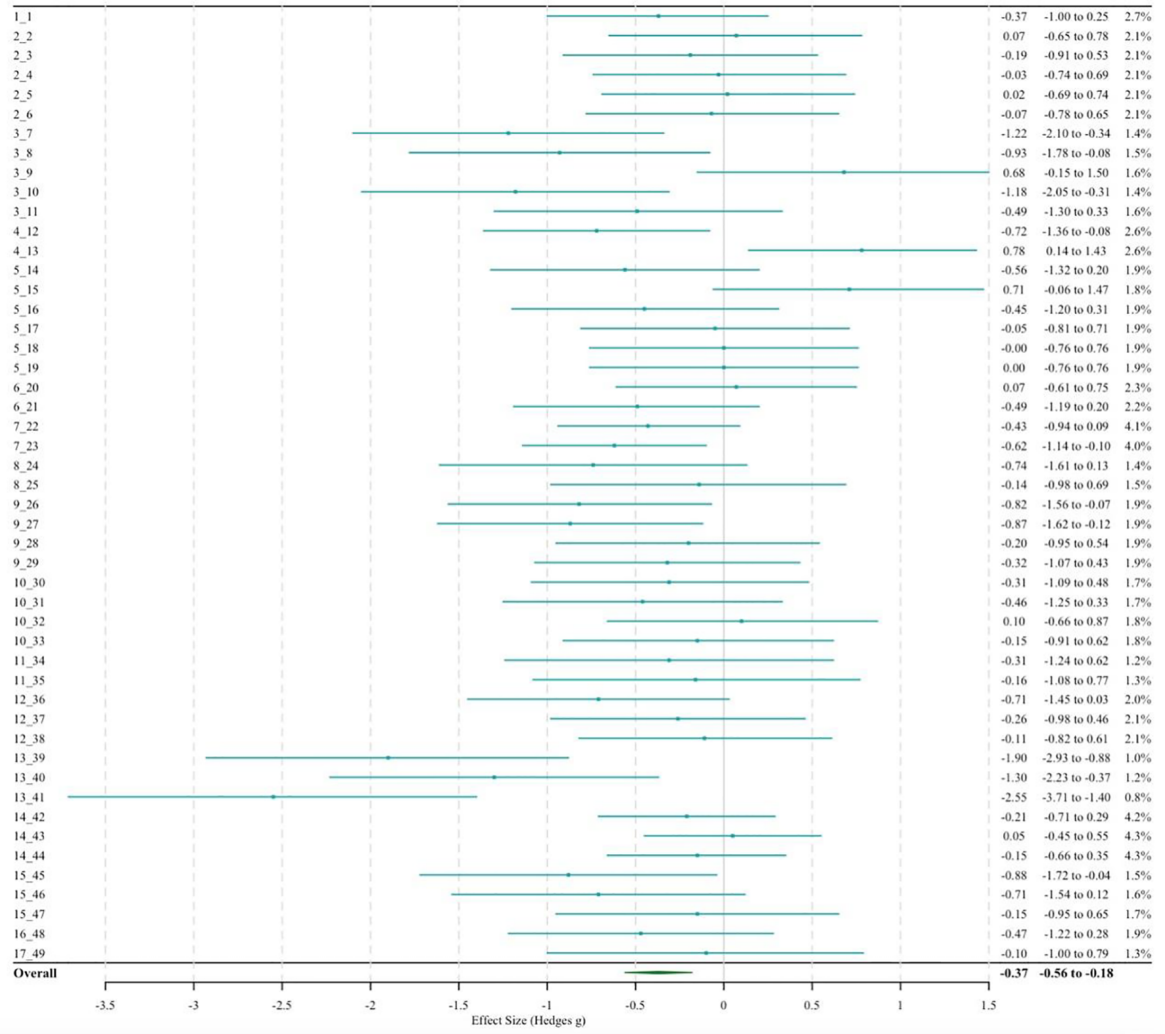
Main effect analysis of exercise interventions on the executive function of children and adolescents.

To evaluate the overall impact of exercise interventions on EF, all effect sizes were pooled using a three-level random-effects model (**Figure 2**). The model yielded a moderate and statistically significant effect (*g* = −0.37, 95% CI: −0.56 to −0.18, *p* = 0.0003). A two-level random-effects model produced a highly comparable estimate (*g* = −0.34, 95% CI: −0.48 to −0.20, *p* < 0.0001), supporting the robustness of the finding. Cochran’s Q test indicated significant heterogeneity across studies (*Q* = 85.20, *p* = 0.0008), suggesting meaningful variation in effect sizes.

### 3.5 Sensitivity analysis

**Figure 3:**
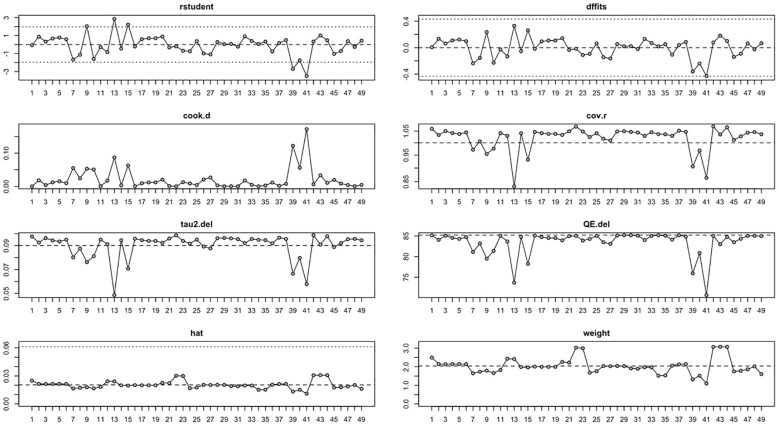
Influence diagnostics for the three-level meta-analysis model.

To evaluate the robustness of the three-level meta-analytic estimates, a sensitivity analysis was performed using standardized residuals (|z| > 3), which identified three potentially anomalous effect sizes (effect_id = 13, 39, 41; see **Figure 3**). After excluding these influential effects, the three-level model was re-estimated. The pooled effect size showed slight attenuation but remained statistically significant (*g* = −0.34, 95% CI: −0.47 to −0.20, *p* < 0.0001), and the prediction interval narrowed substantially (95% PI: −0.63 to −0.037), indicating enhanced stability of the estimate. Results from the two-level model were similarly consistent (*g* = −0.32, 95% CI: −0.43 to −0.20, *p* < 0.001).

Importantly, removing these effect sizes led to a marked reduction in heterogeneity (Q = 50.42, *p* = 0.268), and the within-study variance component (σ^2^_effect) approached zero, suggesting that much of the initial heterogeneity originated from these anomalous contributions. Although exclusion of the influential effects did not change the direction or statistical significance of the pooled effect, it produced a more coherent heterogeneity and variance structure, thereby strengthening the robustness of the findings. Accordingly, the cleaned dataset was used for subsequent analyses of overall and moderating effects to ensure the reliability of the study’s conclusions.

### 3.6 Heterogeneity analysis

To further clarify the sources of heterogeneity, a three-level random-effects model was used to decompose the total variance after excluding outliers, following the framework proposed by Cheung (25). The variance decomposition indicated that Level 1 sampling error accounted for the largest proportion (*I^2^* = 88.44%), suggesting that most variability among effect sizes arose from sampling fluctuation. Level 2 within-study variance was negligible (*I^2^* ≈ 0%), whereas Level 3 between-study variance accounted for 11.56% of the total variance.

We then evaluated the necessity of each variance component using LRTs. First, a two-level model with Level 2 variance fixed at zero was compared with the full three-level model. The two models showed identical fit (LRT = 0.00, *p* = 1.000), confirming that within-study variance did not contribute meaningfully to model performance. Second, when Level 3 variance was set to zero and the model was refitted, the simplified model again did not significantly differ from the three-level model (LRT = 1.22, *p* = 0.270), although the between-study component accounted for 11.56% of the heterogeneity. Statistically, these results indicate no significant improvement in model fit when moving from a two-level to a three-level structure. However, the data inherently exhibit a hierarchical structure, with multiple effect sizes nested within studies. Many trials reported several EF outcomes, thereby creating statistical dependence that, if ignored, may underestimate standard errors and inflate statistical significance. Therefore, to prioritize methodological rigor and stable inference, this study adopted a three-level random-effects model for the estimation of overall effects, subgroup analyses, and meta-regression. This approach more accurately represents the data structure and appropriately accounts for correlations among effect sizes.

### 3.7 Moderator analysis

#### 3.7.1 Subgroup analysis

As shown in **Table 4**, Subgroup analyses indicated that intervention frequency, duration, single-session duration, intervention type, and total intervention dose were generally associated with significant improvements in EF; however, only intervention duration demonstrated significant between-group differences.

**Table 4:** Subgroup analysis of the effects of exercise intervention on the executive function of children and adolescents.

Subgroup	k	g	95% CI	P	F	df	Level 2 I²	Level 3 I²
Frequency				0.951	0.051	2	16.54%	<1%
1-2/week	14	-0.35	-0.58, -0.11	0.005				
3-4/week	19	-0.36	-0.58, -0.14	0.002				
4-5/week	13	-0.39	-0.59, -0.02	0.04				
Duration *				0.049	3.238	2	<1%	2.95%
≤4week	16	-0.37	-0.57, -0.16	0.001				
6-8week	17	-0.15	-0.34,0.03	0.096				
≥10week	13	-0.5	-0.71, -0.29	<0.001				
Exercise session time				0.787	0.241	2	14.39%	<1%
<40min	13	-0.38	-0.64, -0.12	0.006				
40-55min	13	-0.38	-0.64, -0.13	0.043				
≥60min	20	-0.29	-0.49, -0.09	0.062				
Intervention				0.233	1.485	3	<1%	9.25%
MPA	27	-0.36	-0.54, -0.17	<0.001				
AE	5	-0.12	-0.50, 0.26	0.534				
MBE	7	-0.20	-0.46, 0.06	0.122				
Exergaming	7	-0.53	-0.81, -0.25	<0.001				
Total intervention time				0.292	1.268	2	<1%	9.01%
≤600min	20	-0.29	-0.49, -0.09	0.048				
600-1200min	14	-0.26	-0.48, -0.03	0.025				
>1200min	12	-0.50	-0.76, -0.25	0.002				
Domain				0.257	1.407	2	<1%	10.02%
Inhibitory control	15	-0.46	-0.66, -0.26	<0.001				
Working memory	9	-0.24	-0.50, 0.03	0.078				
Cognitive flexibility	20	-0.28	-0.46, -0.10	0.004				

k represents the number of effect sizes. AE aerobic exercise, MBE mind–body exercise, MPA multicomponent physical activity.

The symbol * indicates a statistically significant subgroup difference.

Intervention frequency. Significant effects were observed across all frequency levels, including 1–2 sessions per week (*g* = −0.35, 95% CI: −0.58 to −0.11, *p* = 0.005), 3–4 sessions per week (*g* = −0.36, *95% CI*: −0.58 to −0.14, *p* = 0.002), and 4–5 sessions per week (*g* = −0.39, 95% CI: −0.59 to −0.02, *p* = 0.040). However, no significant differences emerged among frequency groups (*F* = 0.051, *df* = 2, *p* = 0.951).

Intervention duration. Both ≤ 4 weeks (*g* = −0.37, 95% CI: −0.57 to −0.16, *p* = 0.001) and ≥ 10 weeks (*g* = −0.50, 95% CI: −0.71 to −0.29, *p* < 0.0001) produced significant improvements. In contrast, the 6–8-week subgroup did not reach statistical significance (*g* = −0.15, 95% CI: −0.34 to 0.03, *p* = 0.096). Importantly, differences across duration groups were statistically significant (*F* = 3.238, *df* = 2, *p* = 0.049), indicating that intervention duration may be a key determinant of efficacy, with programs lasting ≥ 10 weeks yielding the strongest effects.

Exercise session time. Interventions lasting 40–55 minutes showed significant benefits (*g* = −0.38, 95% CI: −0.64 to −0.13, *p* = 0.004). Effects for < 40 minutes (*g* = −0.38, 95% CI: −0.64 to −0.12, *p* = 0.006) and ≥ 60 minutes (*g* = −0.29, 95% CI: −0.49 to −0.09, *p* = 0.006) were also statistically significant. No significant differences emerged among session-duration groups (*F* = 0.241, *df* = 2, *p* = 0.787).

Total intervention time. Significant effects were observed across all dosage levels, including ≤ 600 minutes (*g* = −0.29, 95% CI: −0.49 to −0.09, *p* = 0.048), 600–1200 minutes (*g* = −0.26, 95% CI: −0.48 to −0.03, *p* = 0.025), and ≥ 1200 minutes (*g* = −0.50, 95% CI: −0.76 to −0.25, *p* = 0.002). However, no significant differences were observed among dosage groups (*F* = 1.268, *df* = 2, *p* = 0.292).

Intervention type. Multicomponent physical activity (MPA) (*g* = −0.36, 95% CI: −0.54 to −0.17, *p* < 0.001) and exergaming (*g* = −0.53, 95% CI: −0.81 to −0.25, *p* < 0.001) demonstrated significant improvements in EF. In contrast, mind–body exercise (*g* = −0.20, 95% CI: −0.46 to 0.06, *p* = 0.122) and aerobic exercise (*g* = −0.12, 95% CI: −0.50 to 0.26, *p* = 0.534) did not reach statistical significance. No significant differences were observed among intervention types (*F* = 1.485, *df* = 3, *p* = 0.233).

EF domain. It should be noted that the following subgroup analyses across EF domains were conducted after excluding two studies that reported only an overall EF score. Significant effects were observed in two EF subdomains, including inhibitory control (*g* = −0.46, 95% CI: −0.66 to −0.26, *p* < 0.001) and cognitive flexibility (*g* = −0.28, 95% CI: −0.46 to −0.10, *p* = 0.004). In contrast, the effect for working memory did not reach statistical significance (*g* = −0.24, 95% CI: −0.50 to 0.03, *p* = 0.078). However, no significant differences were observed among EF domains (*F* = 1.407, *df* = 2, *p* = 0.257).

Most intervention parameters produced significant improvements in EF; however, only intervention duration demonstrated statistically significant between-group differences, suggesting that the length of the intervention period may be the most influential factor in determining treatment efficacy.

#### 3.7.2 Meta-regression

**Figure 4:**
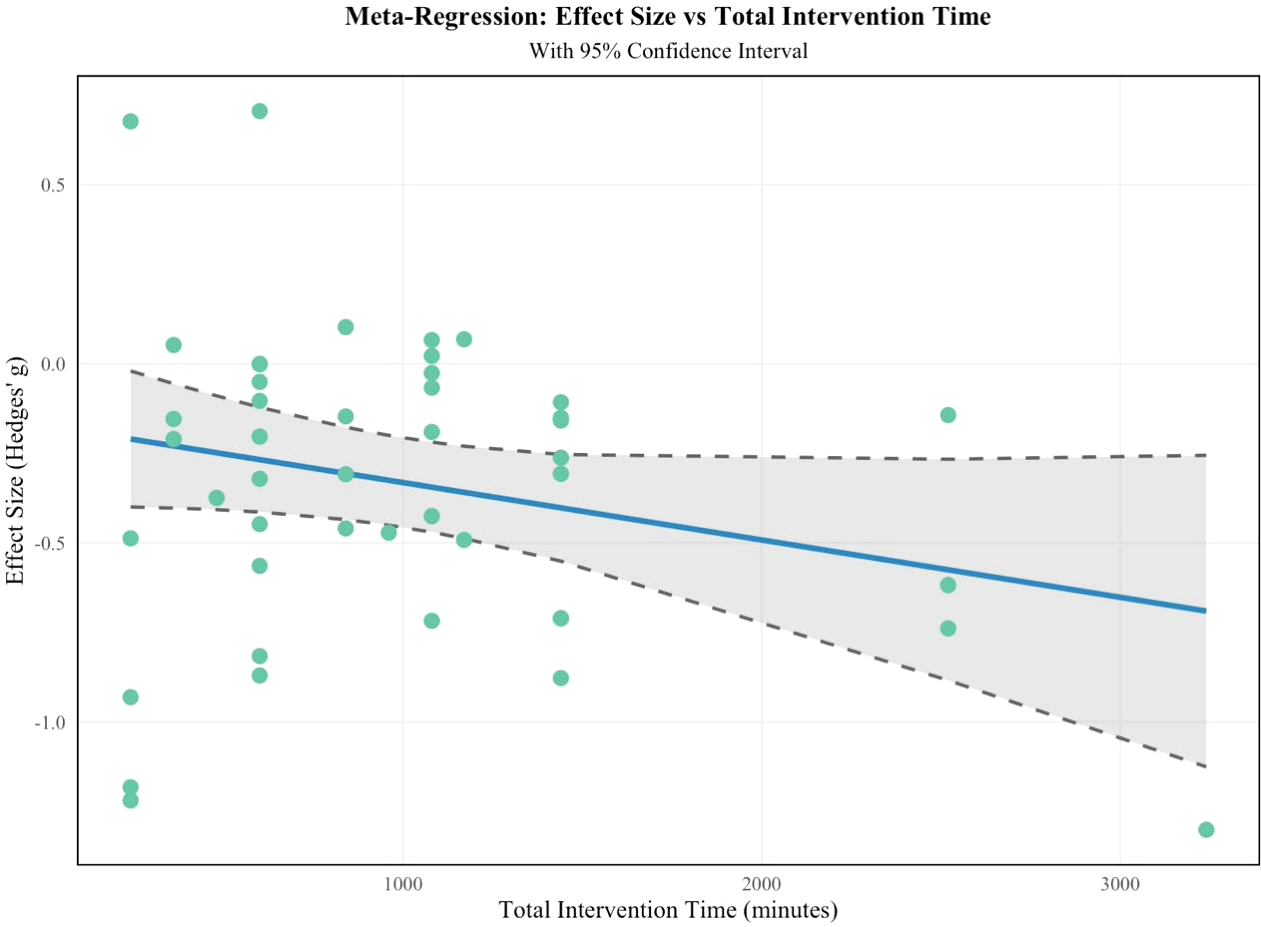
Meta-regression of effect size on total intervention time.

To explore whether a dose–response relationship exists between total intervention duration and improvements in EF, a linear meta-regression was conducted using a three-level random-effects model. The results of the meta-regression analysis are presented in **Figure 4**. The analysis revealed a negative trend between total intervention duration (in minutes) and effect size; however, this association did not reach statistical significance (*F*_<(>1, 44<)>_ = 2.83, *p* = 0.099; *β* = −0.0002, 95% CI: −0.0004 to 0.0000). Heterogeneity decomposition showed low between-study variance (*I^2^*_between = 9.07%) and near-zero within-study variance (*I^2^*_within = 0%), suggesting that total duration accounted for only a small proportion of variability in effects. Overall, although longer interventions may be associated with greater improvements, the current evidence does not support a definitive linear dose–response relationship.

### 3.8 Publication bias

**Figure 5:**
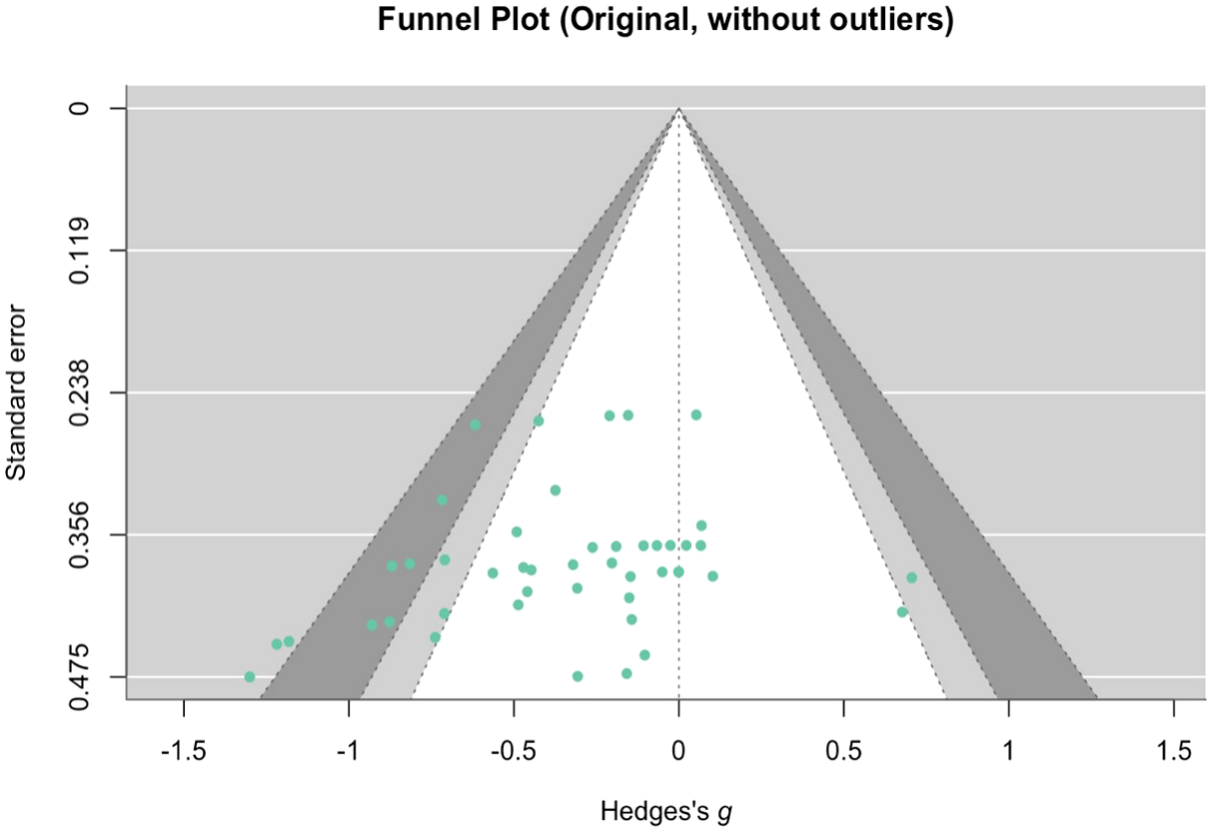
Funnel plot of exercise intervention effects on executive function.

As shown in **Figure 5**, The funnel plot demonstrated an overall symmetrical distribution, with slight deviation among a few studies with larger standard errors; however, this pattern did not indicate systematic asymmetry. Egger’s regression test under the two-level random-effects model was non-significant (*t* = −1.29, *p* = 0.202), and the sample size-adjusted Egger model likewise detected no evidence of asymmetry (*F*_(1, 44)_ = 0.55, *p* = 0.46), suggesting no systematic association between effect size and study precision. The trim-and-fill procedure identified no missing studies (missing = 0), and the pooled effect size remained unchanged after adjustment. Taken together, these complementary analyses indicate an absence of meaningful publication bias or small-sample effects, supporting the robustness of the effect size estimates.

## 4 Discussion

### 4.1 Summary of main findings

This study systematically evaluated the effects of exercise interventions on EF in children and adolescents with ASD using a three-level meta-analytic approach. Analysis of 17 RCTs demonstrated that exercise interventions produced a moderate and statistically significant improvement in EF (*g* = −0.37, 95% CI: −0.56 to −0.18, *p* = 0.0003), supporting exercise as an effective non-pharmacological strategy for managing executive dysfunction. Moderator analyses suggested that intervention duration may be associated with differences in intervention efficacy, with programs lasting ≥ 10 weeks showing relatively more consistent improvements in EF. However, these findings should be interpreted cautiously because the dose–response meta-regression did not reach statistical significance.

### 4.2 Comparison with previous studies

This study demonstrated that exercise interventions significantly improved EF in children and adolescents with ASD, consistent with previous systematic reviews and meta-analyses. Existing evidence indicates that exercise yields small to moderate benefits for overall EF, with more consistent improvements observed in inhibitory control and cognitive flexibility, whereas findings for working memory remain mixed (19, 21). However, conclusions regarding intervention dosage differ from the present findings, as prior evidence has shown no significant differences across exercise characteristics (20). In contrast, this study further suggested that intervention duration may represent a statistically significant moderating factor associated with variability in EF outcomes, with exercise programs lasting ≥ 10 weeks tending to show relatively more consistent improvements in EF. Such inconsistencies are likely related to methodological differences among reviews. Most earlier meta-analyses relied on conventional two-level models and simplified the treatment of multiple outcomes within the same study, which may have obscured statistical dependencies among effect sizes. In contrast, the present study applied a three-level meta-analytic framework. Although likelihood ratio tests indicated only limited improvements in model fit, the three-level structure was retained because multiple effect sizes were nested within studies and modeling these dependencies was considered methodologically more appropriate. In addition, only randomized controlled trials were included, thereby allowing the findings to be based on a higher-quality evidence base and improving the interpretability of the pooled estimates.

### 4.3 Moderating effects and heterogeneity of exercise interventions

Subgroup analyses indicated that most exercise prescription variables, including intervention frequency, session duration, intervention type, and total intervention time, did not demonstrate statistically significant between-group differences, whereas only intervention duration demonstrated a significant moderating effect.

Regarding intervention duration, this study categorized exercise intervention durations into three groups: ≤ 4 weeks, 6–8 weeks, and ≥ 10 weeks. The results indicated that both the ≤ 4-week and ≥ 10-week groups demonstrated significant effects, whereas the 6–8-week group did not reach statistical significance. Collectively, these findings suggest a pattern in which shorter and longer intervention periods were associated with greater effectiveness than intermediate durations. This pattern may be related to both short-term adaptive responses and long-term cumulative effects of exercise. On the one hand, short-term exercise may induce immediate improvements in EF through state-dependent neurophysiological regulation, including transient increases in catecholamines and brain-derived neurotrophic factor (BDNF), together with enhanced prefrontal activation and cerebral hemodynamic responses (52–56). On the other hand, long-term exercise may improve EF in children and adolescents with ASD through more stable structural and functional brain adaptations induced by sustained intervention, including enhanced neuroplasticity, improved neural network efficiency, and increased white matter integrity (36, 57–60).

Regarding intervention frequency, all frequency groups demonstrated varying degrees of improvement in EF; however, the between-group differences did not reach statistical significance. This finding suggests that simply increasing intervention frequency may not consistently produce greater benefits for EF. One possible explanation is that intervention frequency alone may not adequately reflect the actual cognitive demands embedded within each training session. Compared with repeatedly performing exercise interventions with relatively low cognitive load, programs involving greater cognitive engagement may more effectively activate neural mechanisms related to EF through processes such as response inhibition, attentional shifting, and task switching, even when delivered at a lower frequency.

Regarding exercise session duration, interventions with session lengths of < 40 minutes and 40–55 minutes both demonstrated significant effects, whereas the ≥ 60-minute group showed an improvement trend but did not reach statistical significance. This finding may be related to the cognitive and behavioral characteristics of children and adolescents with ASD. Compared with moderate session durations, excessively long exercise sessions may increase cognitive fatigue and attentional resource depletion, thereby reducing sustained engagement and potentially weakening the effectiveness of the intervention (61).

Regarding intervention type, exergaming and MPA appear to be more effective in improving EF in children and adolescents with ASD. The benefits of exergaming may stem from the substantial enhancement in enjoyment and motivation experienced during physical activity. Compared with traditional aerobic exercise, exergaming may provide a more enjoyable physical activity experience by incorporating interactive challenges, feedback, and reward mechanisms (62). From the perspective of educational and motor learning theory, this environment—characterized by rich content, rapid feedback, and timely rewards—may increase engagement among children and adolescents with ASD and reinforce learning outcomes. The effectiveness of MPA may be associated with its higher level of cognitive engagement and more complex task organization. Compared with single-format physical activities, MPA requires continuous attentional regulation, behavioral adaptation, and response inhibition within dynamic task contexts, while also integrating multiple movement forms and task demands. These high cognitive-load characteristics may provide greater stimulation of EF-related processes and may more effectively engage neural systems associated with higher-order cognitive functioning, thereby contributing to more pronounced improvements in EF (63).

Regarding EF domains, the findings indicated that exercise interventions significantly improved inhibitory control and cognitive flexibility, whereas no statistically significant improvement was observed for working memory. This discrepancy may be related to the inherent characteristics of exercise interventions. Most exercise interventions naturally involve rule-based constraints, dynamic environments, and continuous behavioral regulation, requiring individuals to inhibit inappropriate responses, allocate attentional resources, and flexibly adapt behavior according to changing task demands. These processes closely correspond to the core functions of inhibitory control and cognitive flexibility, which may explain why these domains appear to be more sensitive to exercise interventions.

In contrast, exercise interventions did not demonstrate a statistically significant improvement in working memory. This finding may be explained by the fact that most of the included exercise programs primarily emphasized immediate responses and behavioral adaptation to environmental changes while placing relatively limited demands on the core processes of working memory, including the continuous maintenance, processing, and updating of information. In addition, the relatively small number of studies assessing working memory, together with variability in measurement tools and task paradigms across studies, may have contributed to the instability and heterogeneity of the findings.

This study conducted subgroup analyses and meta-regression to examine the potential linear relationship between exercise intervention and total intervention dose. The meta-regression showed a positive trend between total intervention duration and improvements in EF; however, this association did not reach statistical significance (*p* = 0.099). Therefore, this result should be interpreted with caution and cannot be considered reliable evidence of a dose–response relationship. Rather, it represents a preliminary signal that may be sensitive to sampling variability and limited statistical power. Although the subgroup analyses showed similar tendencies, these findings remain exploratory. Larger samples and more rigorously designed trials are required to confirm whether a true dose–response relationship exists and to determine the optimal dosage range.

It should be noted that although heterogeneity was addressed through the use of random-effects models and other statistical approaches, substantial variability remained among the included exercise interventions in terms of exercise modality, intervention dosage, and implementation format. Such heterogeneity may affect the stability of the effect sizes and, to some extent, limit the generalizability and applicability of the findings. For example, different forms of exercise may not influence EF through identical mechanisms. Some activities place greater emphasis on rule switching, task shifting, and sustained attentional regulation, whereas others rely more heavily on repetitive movement practice. Therefore, even when exercise interventions are categorized into broader intervention types, heterogeneity may still exist within categories, potentially obscuring the true effects of specific exercise modalities. In addition, differences in intervention dosage across studies may further limit the transferability of the findings to populations with different ages, functional levels, and educational or clinical contexts. Therefore, future research should further standardize the reporting of exercise prescription parameters and examine the independent and interactive effects of different dosage factors, while also considering intervention type and cognitive load characteristics, in order to identify more precise and targeted exercise intervention strategies.

### 4.4 Neurocognitive mechanisms of exercise intervention effects on EF

The core mechanism by which exercise interventions improve EF in children and adolescents with ASD may stem from their promotion of brain structural plasticity and functional network efficiency. Previous research suggests that EF deficits in ASD are associated with delayed prefrontal development and atypical connectivity patterns, including local hyperconnectivity and insufficient long-range connectivity, which may contribute to network imbalances (64, 65). Therefore, the effects of exercise interventions on EF may involve multiple interacting neurobiological mechanisms.

At the level of neural architecture, exercise interventions may increase metabolic demands and cerebral blood flow perfusion, thereby promoting the production and release of brain-derived neurotrophic factor (BDNF) (66). Upregulation of BDNF has been associated with neuroplasticity and synaptic adaptation (63, 67, 68), which may provide a neural foundation for EF improvement.

At the level of brain functional organization, exercise interventions may contribute to EF improvement by promoting more efficient functional connectivity and cognitive resource allocation. Previous studies indicate that individuals with ASD commonly exhibit cortical connectivity abnormalities and insufficient recruitment of the prefrontal cortex during cognitive processing (69, 70). Regular exercise has been associated with functional network reorganization (71), and neuroimaging studies further suggest that exercise interventions may enhance connectivity among executive control–related brain regions, including the medial prefrontal cortex and posterior cingulate cortex (72, 73). In addition, exercise may modulate activation states across multiple brain regions, including the frontal association cortex, precentral gyrus, lingual gyrus, cingulate gyrus, parietal gyrus, caudate nucleus, and cerebellar vermis, which may support more efficient attentional allocation and cognitive processing (74). Neurophysiological evidence further indicates that exercise may increase P3 amplitude and shorten P3 latency, suggesting improved information processing efficiency and cognitive resource utilization (75).

In summary, exercise interventions may improve EF in children and adolescents with ASD by promoting both neuroplasticity and functional network efficiency within executive control systems.

### 4.5 Clinical application value and implementation recommendations

Based on the findings of this study, several recommendations can be proposed for clinical and educational practice. With regard to the type of exercise intervention, priority should be given to activities involving a high degree of cognitive engagement, such as exergaming and MPA. Such exercise interventions not only enhance the willingness of children and adolescents with ASD to participate but also demonstrate more pronounced effects in improving EF. Regarding intervention dosage, subgroup analyses indicate that a regimen lasting ≥ 10 weeks, comprising 3–5 sessions per week, with each session not exceeding 55 minutes, may be more conducive to achieving favorable intervention outcomes. However, these findings should be interpreted cautiously and may serve primarily as exploratory reference points for exercise dosage arrangements in clinical and educational settings. In addition, based on the findings of this study and relevant theoretical considerations, the level of cognitive engagement embedded in physical tasks may also be a key factor influencing improvements in EF. Therefore, future clinical and educational practice should not only focus on appropriate dosage arrangements but also emphasize the cognitive demands of physical tasks and the quality of participatory experience, thereby more effectively promoting the development of EF in children and adolescents with ASD.

### 4.6 Research limitations and future directions

Several limitations should be noted. First, most of the included studies lacked detailed reports on participant characteristics, such as ASD subtypes, functional levels, intellectual ability, and comorbidities, which limited the assessment of population heterogeneity. Second, the results of the PEDro scale assessment indicate that the included studies exhibited certain methodological shortcomings, primarily related to inadequate blinding. In particular, blinding of participants and therapists was largely not achieved, which may increase the risk of bias in the study results. On the one hand, the lack of participant blinding may give rise to expectancy effects, thereby potentially overestimating the effectiveness of the intervention (76). On the other hand, the absence of therapist blinding may lead to unintentional guidance or variations in intervention intensity during implementation, thereby affecting the objectivity of the results (77). Furthermore, failure to implement assessor blinding in some studies may also increase the risk of detection bias, particularly when subjective scoring or behavioral assessment measures are involved. Finally, the current body of research remains relatively limited, and some studies have small sample sizes, which may lead to unstable effect estimates, particularly in subgroup analyses.

Therefore, future research should be strengthened in several key areas. First, more high-quality, large-scale, multicenter randomized controlled trials are needed to improve the robustness and generalizability of the evidence. Second, methodological rigor should be enhanced through the adoption of standardized intervention protocols, unified implementation procedures, and comprehensive therapist training to minimize bias during intervention delivery. In situations in which complete blinding is difficult to achieve, future studies should, whenever possible, incorporate objective outcome measures, such as digital assessments or neurophysiological indicators, together with independent assessors and independent data analysis, in order to reduce detection bias associated with subjective judgment and enhance the objectivity and reliability of findings. Third, subgroup analysis protocols should be predefined, and key components of exercise prescriptions should be reported in a more standardized manner to improve the interpretability of results and further clarify potential dose–response relationships. Finally, greater attention should be given to heterogeneity among individuals with ASD. Stratified analyses based on factors such as genetic background, comorbidities, cognitive level, and sex may help facilitate the development of more precise and individualized exercise intervention strategies for children and adolescents with ASD.

## 5 Conclusion

This three-level meta-analysis demonstrates that exercise interventions produce a moderate and statistically significant improvement in EF in children and adolescents with ASD. Among the intervention characteristics examined, intervention duration appeared to be associated with variability in EF outcomes across studies, with programs lasting ≥ 10 weeks showing relatively more consistent improvements in EF, whereas differences across intervention frequency, session duration, and exercise modality did not demonstrate statistically significant subgroup differences. Together, these findings indicate that exercise interventions are associated with improved EF in children and adolescents with ASD and may provide useful evidence to inform exercise prescription in clinical and educational contexts.

## Data Availability

The raw data supporting the conclusions of this article will be made available by the authors, without undue reservation.
