# Supplementary material for: Effects of exercise interventions on executive function in autism spectrum disorder: a three-level meta-analytic review
Source: Front Psychiatry. 2026 Jul 20;17:1780503. doi: 10.3389/fpsyt.2026.1780503 (PMC13430442; doi:10.3389/fpsyt.2026.1780503)
Supplement: Supplementary file 1 [file Table1.docx]

**Supplementary Material 1**

| **Database** | **Search strategy** | **Results** |
| --- | --- | --- |
| **PubMed** | (("Autism Spectrum Disorder"[MeSH Terms] OR "autism spectrum disorder*"[tiab] OR ASD[tiab] OR "autistic disorder*"[tiab] OR "autistic spectrum disorder*"[tiab] OR "disorder, autistic spectrum"[tiab] OR Asperger*[tiab] OR "Asperger disorder*"[tiab] OR "Asperger syndrome*"[tiab] OR "Asperger disease*"[tiab] OR "Asperger's disorder*"[tiab] OR "Asperger's syndrome*"[tiab] OR "childhood disintegrative disorder"[tiab] OR CDD[tiab] OR "Kanner* syndrome"[tiab] OR "infantile autism"[tiab] OR "early infantile autism"[tiab] OR "autism, infantile"[tiab] OR "autism, early infantile"[tiab])AND("executive function*"[tiab] OR "executive control"[tiab] OR inhibition[tiab] OR "inhibitory control"[tiab] OR "response inhibition"[tiab] OR "go/no-go"[tiab] OR "go no go"[tiab] OR GNG[tiab] OR stroop[tiab] OR "stroop task"[tiab] OR "cognitive flexibility"[tiab] OR shifting[tiab] OR "set shifting"[tiab] OR "task switching"[tiab] OR "set switching"[tiab] OR WCST[tiab] OR "wisconsin card sorting test"[tiab] OR "working memory"[tiab] OR WM[tiab] OR "n-back"[tiab] OR "n back"[tiab] OR nback[tiab] OR "digit span"[tiab] OR "digit span forward"[tiab] OR "digit span backward"[tiab])AND(child*[tiab] OR adolescen*[tiab] OR pediatric*[tiab] OR paediatric*[tiab] OR preschool*[tiab] OR "school-age*"[tiab] OR youth[tiab] OR teen*[tiab])AND("Exercise"[MeSH Terms] OR exercise[tiab] OR "physical activit*"[tiab] OR "exercise therap*"[tiab] OR "motor activit*"[tiab] OR "physical training"[tiab] OR "fitness training"[tiab] OR "movement therap*"[tiab] OR rehabilitation[tiab] OR sports[tiab] OR "aerobic exercise"[tiab] OR "resistance training"[tiab] OR "strength training"[tiab] OR "endurance training"[tiab] OR "balance training"[tiab] OR swimming[tiab] OR running[tiab] OR cycling[tiab] OR "jump rope"[tiab] OR "ball game*"[tiab] OR basketball[tiab] OR soccer[tiab] OR football[tiab] OR volleyball[tiab] OR "table tennis"[tiab] OR "martial art*"[tiab] OR judo[tiab] OR karate[tiab] OR taekwondo[tiab] OR "kung fu"[tiab] OR dance[tiab] OR yoga[tiab] OR pilates[tiab] OR "tai chi"[tiab] OR qigong[tiab] OR baduanjin[tiab] OR wuqinxi[tiab] OR yijinjing[tiab] OR exergam*[tiab] OR "mind-body exercise"[tiab])  ) | 91 |
| **Web of Science** | TS=("autism spectrum disorder*" OR ASD OR "autistic disorder*" OR "autistic spectrum disorder*" OR "disorder, autistic spectrum" OR Asperger* OR "Asperger disorder*" OR "Asperger syndrome*" OR "Asperger disease*" OR autism* OR "Asperger's disorder*" OR "Asperger's syndrome*" OR "childhood disintegrative disorder" OR CDD OR "Kanner* syndrome" OR "infantile autism" OR "early infantile autism")ANDTS=("executive function*" OR "executive control" OR inhibition OR "inhibitory control" OR "response inhibition" OR "go/no-go" OR "go no go" OR GNG OR stroop OR "stroop task" OR "cognitive flexibility" OR shifting OR "set shifting" OR "task switching" OR "set switching" OR WCST OR "wisconsin card sorting test" OR "working memory" OR WM OR "n-back" OR "n back" OR nback OR "digit span" OR "digit span forward" OR "digit span backward")ANDTS=("exercise" OR "physical activit*" OR "exercise therap*" OR "motor activit*" OR "physical training" OR "fitness training" OR "movement therap*" OR rehabilitation OR sports OR "aerobic exercise" OR "resistance training" OR "strength training" OR "endurance training" OR "balance training" OR swimming OR running OR cycling OR "jump rope" OR "ball game*" OR basketball OR soccer OR football OR volleyball OR "table tennis" OR "martial art*" OR judo OR karate OR taekwondo OR "kung fu" OR dance OR yoga OR pilates OR "tai chi" OR qigong OR baduanjin OR wuqinxi OR yijinjing OR exergam* OR "mind-body exercise")ANDTS=(child* OR adolescen* OR pediatric* OR paediatric* OR preschool* OR "school-age*" OR youth OR teen*) | 517 |
| **Embase** | ('autism spectrum disorder'/exp OR 'autism spectrum disorder*':ti,ab OR asd:ti,ab OR 'autistic disorder*':ti,ab OR 'autistic spectrum disorder*':ti,ab OR autism:ti,ab OR 'disorder, autistic spectrum':ti,ab OR asperger*:ti,ab OR 'asperger disorder*':ti,ab OR 'asperger syndrome*':ti,ab OR 'asperger disease*':ti,ab OR 'aspergers disorder*':ti,ab OR 'aspergers syndrome*':ti,ab OR 'childhood disintegrative disorder':ti,ab OR cdd:ti,ab OR 'kanner* syndrome':ti,ab OR 'infantile autism':ti,ab OR 'early infantile autism':ti,ab OR 'autism, infantile':ti,ab OR 'autism, early infantile':ti,ab) AND (child*:ti,ab OR adolescen*:ti,ab OR pediatric*:ti,ab OR paediatric*:ti,ab OR preschool*:ti,ab OR 'school-age*':ti,ab OR youth:ti,ab OR teen*:ti,ab) AND ('exercise'/exp OR exercise:ti,ab OR 'physical activit*':ti,ab OR 'exercise therap*':ti,ab OR 'motor activit*':ti,ab OR 'physical training':ti,ab OR 'fitness training':ti,ab OR 'movement therap*':ti,ab OR rehabilitation:ti,ab OR sports:ti,ab OR 'aerobic exercise':ti,ab OR 'resistance training':ti,ab OR 'strength training':ti,ab OR 'endurance training':ti,ab OR 'balance training':ti,ab OR swimming:ti,ab OR running:ti,ab OR cycling:ti,ab OR 'jump rope':ti,ab OR 'ball game*':ti,ab OR basketball:ti,ab OR soccer:ti,ab OR football:ti,ab OR volleyball:ti,ab OR 'table tennis':ti,ab OR 'martial art*':ti,ab OR judo:ti,ab OR karate:ti,ab OR taekwondo:ti,ab OR 'kung fu':ti,ab OR dance:ti,ab OR yoga:ti,ab OR pilates:ti,ab OR 'tai chi':ti,ab OR qigong:ti,ab OR baduanjin:ti,ab OR wuqinxi:ti,ab OR yijinjing:ti,ab OR exergam*:ti,ab OR 'mind-body exercise':ti,ab) AND ('executive function*':ti,ab OR 'executive control':ti,ab OR inhibition:ti,ab OR 'inhibitory control':ti,ab OR 'response inhibition':ti,ab OR 'go/no-go':ti,ab OR 'go no go':ti,ab OR gng:ti,ab OR stroop:ti,ab OR 'stroop task':ti,ab OR 'cognitive flexibility':ti,ab OR shifting:ti,ab OR 'set shifting':ti,ab OR 'task switching':ti,ab OR 'set switching':ti,ab OR wcst:ti,ab OR 'wisconsin card sorting test':ti,ab OR 'working memory':ti,ab OR wm:ti,ab OR 'n-back':ti,ab OR 'n back':ti,ab OR nback:ti,ab OR 'digit span':ti,ab OR 'digit span forward':ti,ab OR 'digit span backward':ti,ab) AND ('randomized controlled trial'/exp OR 'randomized controlled trial':ti,ab OR randomized:ti,ab OR randomised:ti,ab OR random:ti,ab OR 'random allocation'/exp OR placebo:ti,ab) | 199 |
| **Cochrane** | (MeSH descriptor: [Autism Spectrum Disorder] explode all trees OR (Autistic Spectrum Disorder):ti,ab,kw OR (Autistic Spectrum Disorders):ti,ab,kw OR ASD:ti,ab,kw OR Autistic:ti,ab,kw OR (Disorder, Autistic Spectrum):ti,ab,kw OR (Asperger disorder):ti,ab,kw OR (Syndrome, Asperger):ti,ab,kw OR (Asperger's Disease):ti,ab,kw OR (Asperger's Diseases):ti,ab,kw OR (Aspergers Disease):ti,ab,kw OR (Kanner's Syndrome):ti,ab,kw OR (Kanner Syndrome):ti,ab,kw OR (Autism, Infantile):ti,ab,kw OR "infantile autism":ti,ab,kw OR (Early Infantile Autism):ti,ab,kw) AND (MeSH descriptor: [Child] explode all trees OR child:ti,ab,kw OR children:ti,ab,kw OR adolescent:ti,ab,kw OR pediatric:ti,ab,kw OR preschool:ti,ab,kw) AND (MeSH descriptor: [Exercise] explode all trees OR MeSH descriptor: [Sports] explode all trees OR exercise:ti,ab,kw OR (Physical Activity):ti,ab,kw OR (Physical Activities):ti,ab,kw OR (Exercise Training):ti,ab,kw OR (Training, Exercise):ti,ab,kw OR (Aerobic Exercise):ti,ab,kw OR (Resistance Training):ti,ab,kw OR (Strength Training):ti,ab,kw OR (Endurance Training):ti,ab,kw OR (Balance Training):ti,ab,kw OR (Ball Games):ti,ab,kw OR (movement therapy):ti,ab,kw OR (Mind body exercise):ti,ab,kw OR Yoga:ti,ab,kw OR (Tai Chi):ti,ab,kw OR (Tai Chi Chuan):ti,ab,kw OR (Qi-Gong):ti,ab,kw OR Dance:ti,ab,kw OR Pilates:ti,ab,kw OR Baduanjin:ti,ab,kw OR (Aquatic exercise):ti,ab,kw OR Exergame:ti,ab,kw) AND (MeSH descriptor: [Executive Function] explode all trees OR MeSH descriptor: [Inhibition, Psychological] explode all trees OR MeSH descriptor: [Cognitive Flexibility] explode all trees OR MeSH descriptor: [Memory, Short-Term] explode all trees OR (Executive Function):ti,ab,kw OR (Executive Control):ti,ab,kw OR (inhibitory control):ti,ab,kw OR (response inhibition):ti,ab,kw OR GNG:ti,ab,kw OR Stroop:ti,ab,kw OR (Cognitive Flexibility):ti,ab,kw OR (set shifting):ti,ab,kw OR WCST:ti,ab,kw OR (Working Memory):ti,ab,kw OR (digit span):ti,ab,kw OR (n-back):ti,ab,kw) | 40 |
